# Esthetic Rehabilitation with Direct Composite Veneering: A Report of 2 Cases

**DOI:** 10.1155/2017/7638153

**Published:** 2017-04-18

**Authors:** Kyatham Sowmya, K. S. Dwijendra, V. Pranitha, Konda Karthik Roy

**Affiliations:** ^1^Department of Pedodontics and Preventive Dentistry, MNR Dental College & Hospital, Sangareddy, Telangana, India; ^2^Department of Conservative Dentistry & Endodontics, MNR Dental College & Hospital, Sangareddy, Telangana, India

## Abstract

Esthetic or cosmetic dentistry is one of the main areas of dental practice. Increasing demand of patients for esthetics has resulted in the development of several techniques for restoring the anterior teeth. Composite resin restorations have become an integral part of contemporary restorative dentistry and can be called “star of minimal invasion” due to its conservative concepts. The direct composite veneering allows restoring the tooth in a natural way and preservation of sound tooth structure when compared to indirect restorations. This article presents two case reports of esthetic rehabilitation of anterior teeth using direct composite veneering with two-year follow-up with acceptable outcome.

## 1. Introduction

Esthetics is a branch of philosophy which deals with beauty and the beautiful (Merriam Webster dictionary). The goal of esthetic dentistry should be “bright, beautiful, but believable” [1]. Smile is an important feature of face predicting its attractiveness and need for esthetics will motivate patients to seek dental treatment. Pediatric esthetic dentistry is a branch that deals with maintenance and enhancement of beauty of the mouth of infants and children through adolescence, including those with special health care needs [2]. Various treatment modalities for esthetic rehabilitation exist like microabrasion, direct composite resin restorations or combination of both, indirect composites, crowns, esthetic veneers, and so forth.

Direct composite veneers allow operator to control and evaluate entire procedure from shade selection to final morphology usually in a single appointment. It is most commonly utilized form of veneering [3]. These are often been heralded as a more conservative alternative to porcelain. With the advent of microhybrid and nanohybrid composites, finishing and polishing of these restorations can rival that of porcelain [4]. In 1997, Peumans et al. found 89% success rate of direct composite veneers after 5-year follow-up [5]. Esthetics and durability of composite materials have improved dramatically over years. However, materials have some inherent disadvantages like color instability, polymerization shrinkage, and excessive wear. Constant advancement of resin technology and advent of newer materials have resulted in reduced shrinkage, improved color stability, wear resistance, and biocompatibility [6–8].

Direct composite veneering results in minimal invasion and maximum preservation of sound tooth structure when compared to indirect restorations. These restorations can be easily repaired which is a more conservative and preferable option than replacement. Thus, direct composite resin restorations have become a viable alternative for young patients that require anterior restorative procedures [9, 10]. Here we present two case reports of esthetic rehabilitation of anterior teeth in adolescence using direct composites with follow-up of 2 years.

## 2. Case Report 1

**Figure 1:**
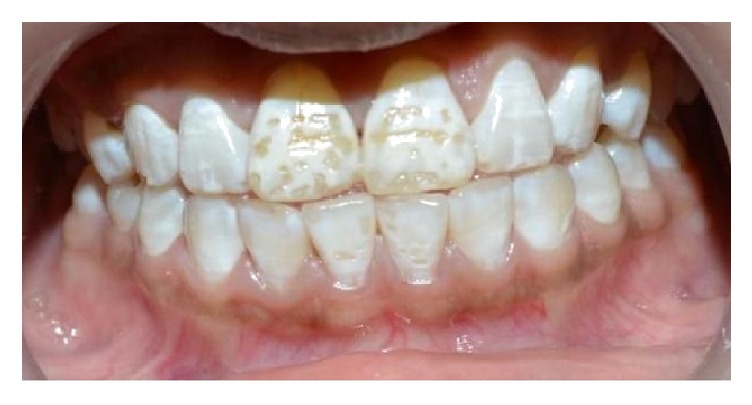
Pretreatment photograph showing severely pitted appearance.

A 14-year-old female child was referred to the Department of Pedodontics and Preventive Dentistry in MNR Dental College & Hospital for the esthetic rehabilitation. The patient was not satisfied with her smile because of the discolored and pitted appearance of maxillary anterior teeth (Deans Fluorosis Index-4, Figure 1). Various treatment options were explained to patient and it was decided to opt for direct composite veneering taking age of patient into consideration.

**Figure 2:**
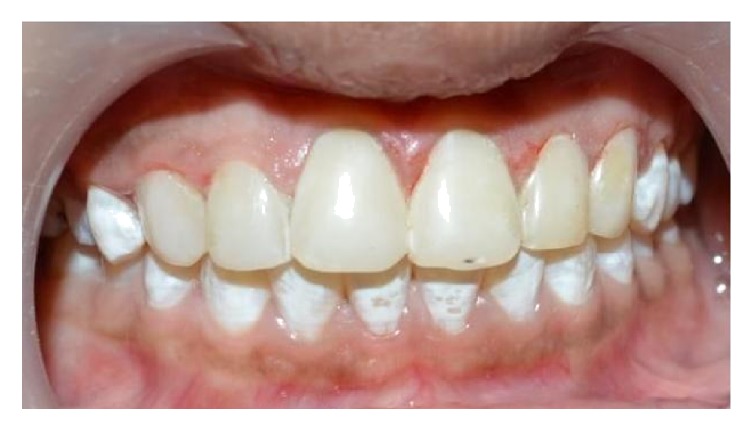
Finished and polished restoration.

**Figure 3:**
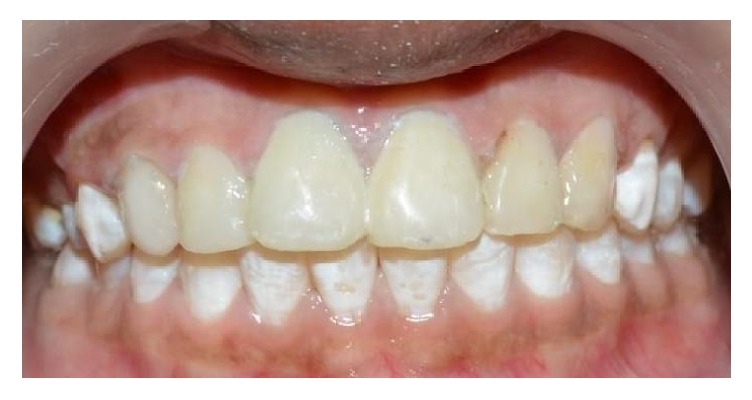
2-year follow-up.

The color was recorded using the VITA Classical shade guide, and shades A1 and A2 were considered as the initial color. The tooth preparation involved a minimal chamfer on the facial surfaces. Cotton rolls and salivary ejectors were used for field isolation. The enamel surface was acid etched using 37% phosphoric acid (D-Tech Etching Gel, Kerr, USA) for 30 seconds (additional etching time of 20 sec because teeth are affected with severe fluorosis) [11], rinsed for 10 seconds, and dried. A bonding agent (Adper Single Bond, 3M ESPE, USA) was applied on the prepared enamel and light-cured for 10 seconds. A stratified layering technique was used to fill the tooth with Filtek™ Z350 XT (3M ESPE, USA) shade A1E. The composite was light-cured for 10 seconds on each surface. Each surface was polymerized from incisal/occlusal, facial, and lingual aspects for additional 20 seconds. Restoration was finished and polished with polishing discs (Ultra Gloss Composite Polishing System, Axis, USA) (Figure 2). Patient was motivated for maintenance of oral hygiene and informed for regular recalls. Recall check-up was done every 6 months and there was no sensitivity or discoloration detected. At 2-year recall check-up, mild debris was noted on restorations (Figure 3) and oral prophylaxis was done and the restorations were finished and polished.

## 3. Case 2

**Figure 4:**
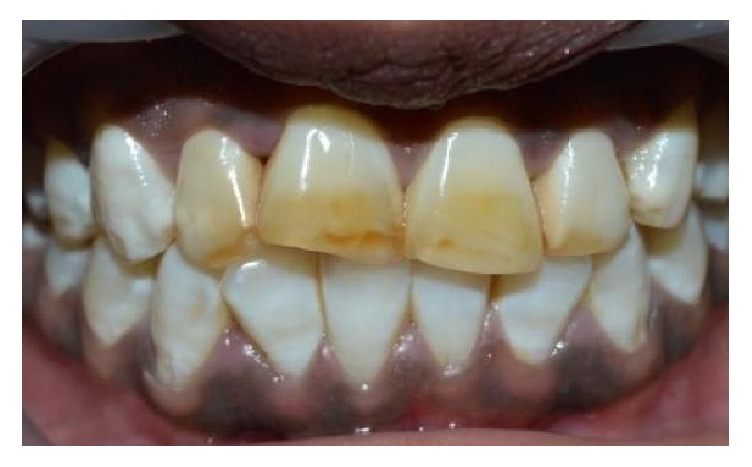
Pretreatment photograph showing discolored teeth.

**Figure 5:**
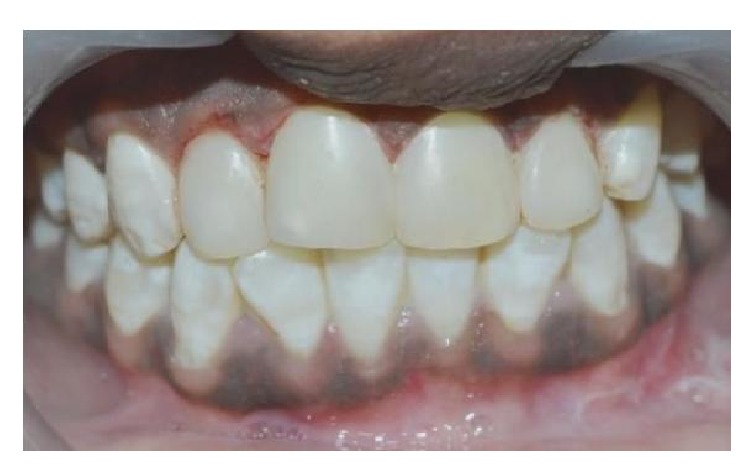
Finished and polished restoration.

**Figure 6:**
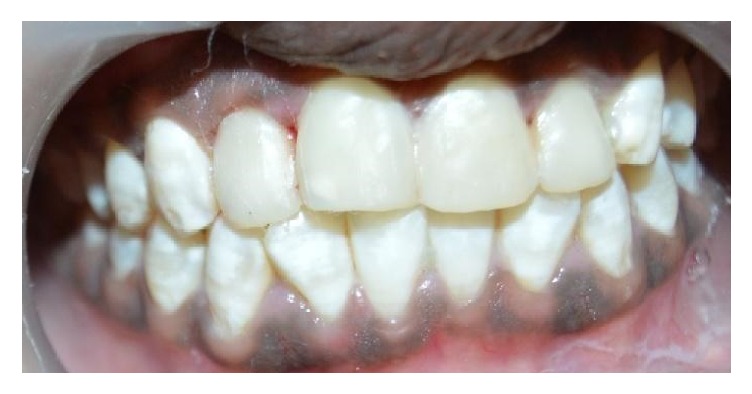
2-year follow-up.

A 15-year-old female patient reported to Department of Pedodontics and Preventive Dentistry with chief complaint of discolored maxillary incisors. On examination, there was slight distolabial rotation of right maxillary central incisor (Figure 4). The treatment plan was to perform direct composite veneering on anterior teeth. The initial shade was measured with the aid of a VITA shade guide (VITAPAN Classical). The tooth preparation involved chamfer on the facial surfaces. The surface was acid etched with 37% phosphoric acid (D-Tech Etching Gel, Kerr, USA) for 15 s, rinsed for 20 s, and dried. A bonding agent (Adper Single Bond, 3M ESPE, USA) was applied on the prepared enamel and light-cured for 10 seconds. Increments of enamel shade composite A1E (Filtek Z350 XT, 3M ESPE, St. Paul, MN, USA) were placed for rebuilding the enamel and photo activated for 20 seconds. The incisal adjustment was performed with fine and extrafine flame-shaped diamond burs, 3168 F and 3168 FF (KG Sorensen, Brazil). Finishing and polishing were performed (Figure 5). The patient was instructed to maintain the oral hygiene and recall visits were scheduled every 6 months. No discolorations or disintegrations were observed at the end of 2-year follow-up (Figure 6).

## 4. Discussion

The importance for esthetics is increasing in the practice of modern pediatric dentistry. The demand for esthetic motivates the patient to seek dental treatment which is often dictated by cultural, ethnic, and individual preferences [11]. The aim of treatment for both the cases is to restore patient's esthetics and self-esteem. Cognitive theory by Jean Piaget states that adolescents are egocentric. This dwelling of one's self may make an individual overly self-conscious [12, 13]. So, esthetics should be taken as a major consideration while treating pediatric patients.

In case 1, the patient had severe pitted anterior teeth which necessitated treatment with veneering procedure. In case 2, patient had discolored anterior teeth and in order to establish both functional and esthetic integrity, veneers were considered.

Direct composite veneers are indicated for esthetic rehabilitation in these cases because of conservative tooth preparation and because they can be completed in single appointment, frequency of replacement or repair is less, they are strong and durable, no luting agent is required, and it is cost-effective. In addition, they have similar abrasion rates as that of natural tooth structures.

Polishing of direct composite veneers is easy and any cracks or fractures on the restoration may be repaired intraorally [14]. Also, marginal adaptation is better than that of indirect veneer restorations [15]. In these cases, the use of conservative direct composite resins provided both symmetrical and harmonious restoration of the teeth. Another point to ponder is that mineral density of Ca and P by weight percentage in outer enamel layer in old age group (≤55 years) is significantly higher than young age group (18–24 years), so conservative minimal invasive techniques should be the treatment of choice in pediatric patients [16].

## 5. Conclusion

It can be concluded that while treating a pediatric patient equal consideration should be paid to esthetics along with function for the proper psychological development of the child.
